# Nutritional Interventions in Cancer Cachexia: Evidence and Perspectives From Experimental Models

**DOI:** 10.3389/fnut.2020.601329

**Published:** 2020-12-22

**Authors:** Wouter R. P. H. van de Worp, Annemie M. W. J. Schols, Jan Theys, Ardy van Helvoort, Ramon C. J. Langen

**Affiliations:** ^1^Department of Respiratory Medicine, NUTRIM–School of Nutrition and Translational Research in Metabolism, Maastricht University Medical Center+, Maastricht, Netherlands; ^2^Department of Precision Medicine, GROW–School for Oncology and Developmental Biology, Maastricht University Medical Center+, Maastricht, Netherlands; ^3^Danone Nutricia Research, Utrecht, Netherlands

**Keywords:** cachexia, cancer, undernutrition, nutrition, multinutrient, multitarget, multimodal

## Abstract

Cancer cachexia is a complex metabolic syndrome characterized by involuntary skeletal muscle loss and is associated with poor clinical outcome, decreased survival and negatively influences cancer therapy. No curative treatments are available for cancer cachexia, but nutritional intervention is recommended as a cornerstone of multimodal therapy. Optimal nutritional care is pivotal in the treatment of cancer cachexia, and the effects of nutrients may extend beyond provision of adequate energy uptake, targeting different mechanisms or metabolic pathways that are affected or deregulated by cachexia. The evidence to support this notion derived from nutritional intervention studies in experimental models of cancer cachexia is systematically discussed in this review. Moreover, experimental variables and readout parameters to determine skeletal muscle wasting and cachexia are methodologically evaluated to allow critical comparison of similar studies. Single- and multinutrient intervention studies including qualitative modulation of dietary protein, dietary fat, and supplementation with specific nutrients, such as carnitine and creatine, were reviewed for their efficacy to counteract muscle mass loss and its underlying mechanisms in experimental cancer cachexia. Numerous studies showed favorable effects on impaired protein turnover and related metabolic abnormalities of nutritional supplementation in parallel with a beneficial impact on cancer-induced muscle wasting. The combination of high quality nutrients in a multitargeted, multinutrient approach appears specifically promising, preferentially as a multimodal intervention, although more studies investigating the optimal quantity and combination of nutrients are needed. During the review process, a wide variation in timing, duration, dosing, and route of supplementation, as well as a wide variation in animal models were observed. Better standardization in dietary design, and the development of experimental models that better recapitulate the etiology of human cachexia, will further facilitate successful translation of experimentally-based multinutrient, multimodal interventions into clinical practice.

## Introduction

Cancer cachexia is a debilitating syndrome characterized by involuntary weight loss that not only affects adipose tissue but also leads to wasting and weakness of skeletal muscle. Cachexia is associated with an abnormal energy and substrate metabolism that cannot be reversed by conventional nutritional support (1). This differentiates the syndrome from (semi)starvation during which energy expenditure and protein turnover is reduced. Cachexia is highly prevalent in advanced cancers. One third of all patients with cancer loses more than 5% of their original body weight, which is a common screening criterion for cancer cachexia (1, 2). In particular, about 70% of gastric cancer patients, 80% of lung cancer patients, and 90% of liver and pancreatic cancer patients are at risk of developing cachexia (2). Cachexia is associated with poor clinical outcome, decreased survival (3) and negatively influences tumor therapy, as is illustrated by increased postoperative mortality and decreased response to radiation-, chemo-, and immunotherapy (4–6). Muscle wasting is an important contributing factor to muscle weakness in cachexia, which adversely affects performance status, quality of life and hospitalization risk of cancer patients (7).

Many factors contribute to cancer-induced weight loss including anorexia, altered protein and energy metabolism, and chronic inflammation. The anorexia associated with cancer cachexia is likely caused by the activity of pro-inflammatory cytokines, such as tumor necrosis factor-α (TNF-α), interleukin-1 (IL-1), interleukin-6 (IL-6) and growth differentiation factor 15, that are produced either by the tumor or by the host in response to the tumor, which interfere with appetite signals within the anterior hypothalamus (8, 9). Some of these cytokines may also increase the metabolic rate in cancer cachexia (10). In addition to hypermetabolism, one of the major metabolic changes contributing to cancer cachexia is the alteration in protein metabolism. These changes are characterized by a net protein breakdown and an increased oxidation of branched-chain amino acids (BCAAs), especially in the skeletal muscle, to support energy production (gluconeogenesis) and to supply amino acids for elevated hepatic synthesis of acute-phase proteins (11). The breakdown of the host protein is partly stimulated by tumor-secreted factors, including proteolysis-inducing factor and lipid-mobilizing factor, and host-derived inflammatory cytokines such as TNF- α and IL-6 (12).

Undernutrition is a common problem in patients with cancer cachexia and can be a consequence of both reduced dietary intake, poor dietary quality and hypermetabolism (13). The reduced food intake is thought to be explained by tumor-induced symptoms, such as mechanical interference with nutritional intake or absorption, treatment-related side effects, including mucositis, nausea and vomiting, and altered taste. Undernutrition not only affects the macronutrients that supply energy but also the micronutrients that are essential cofactors in metabolism and vital to preserve body mass Therefore, undernutrition is one of the main reasons that patients with cancer cachexia have an inadequate micronutrient status (14). Accordingly, inadequate micronutrient intake negatively influences the course of the disease and increases the risk of complications.

**Figure 1:**
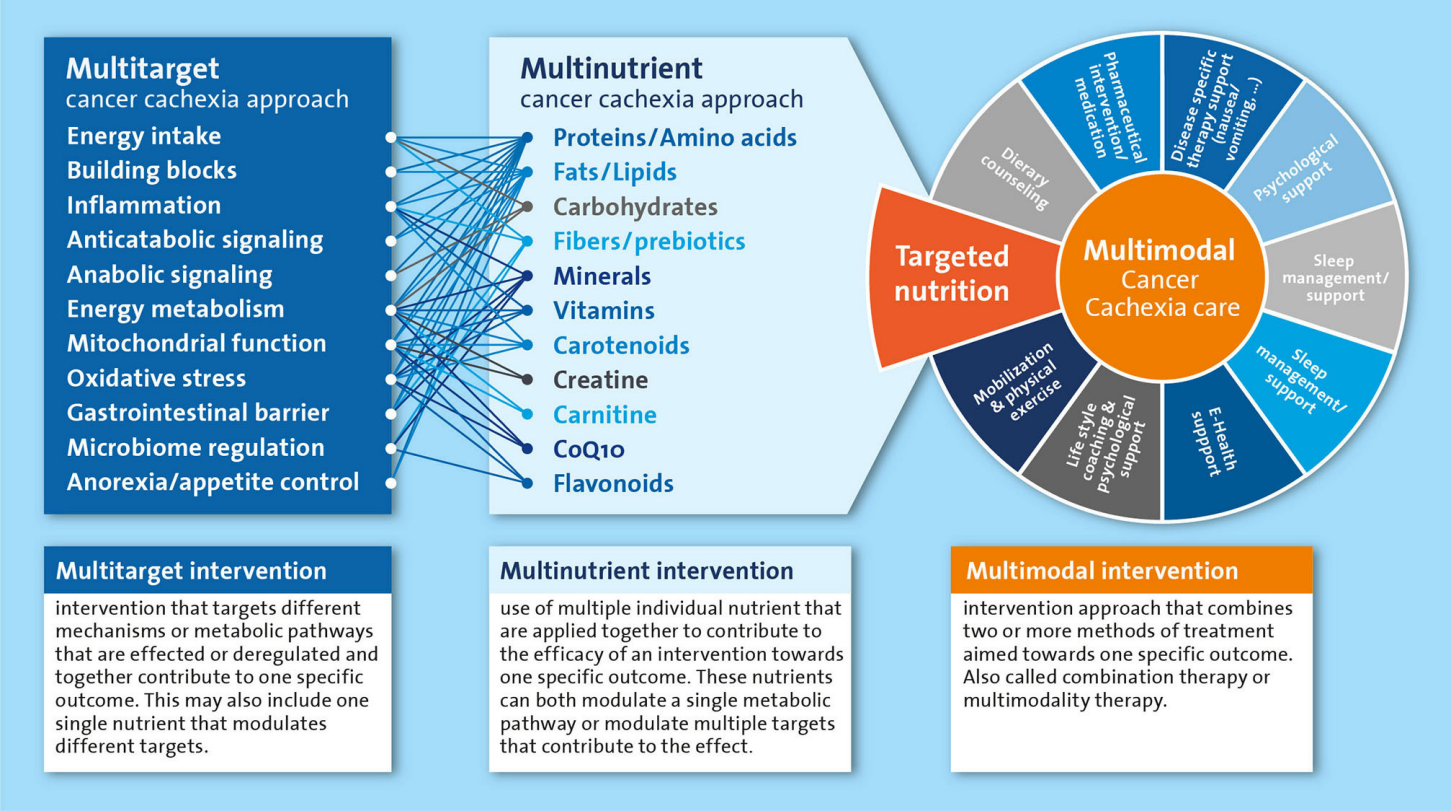
Simplified representation of the multitarget and multinutrient approach and their mutual interactions as part of the multimodal cancer cachexia care. All components listed under “multinutrient cancer cachexia approach” are systematically reviewed as single nutrients or as part of multinutrient approaches in the text.

For a long time, undernutrition and cachexia remained neglected medical conditions for which a clear therapeutic strategy was lacking. A multidisciplinary approach is currently considered the best option to tackle cancer cachexia, in which nutritional intervention is recommended as an integral part of the multimodal therapy (15). Adequate nutritional care is pivotal to provide the essential building blocks to maintain and rebuild tissue in cachectic patients. Furthermore, nutrition is also crucial to supply energy and micronutrients that are vital to fuel and catalyze metabolic processes. In addition to the nourishing of the cachectic patients, various nutrients have been implied in the regulation and normalization of metabolic processes underlying the wasting in cachectic patients. Most of the metabolically active nutrients have different functions in the various intertwined processes that may be deregulated in cachexia. For nutrients and their metabolites that are rate limiting in key metabolic pathways, preventing any deficiencies will help preserve or restore metabolic homeostasis. Furthermore, an approach where multiple nutrients are combined in a well-balanced multi-nutrient intervention is likely most appropriate, as it is well-recognized that nutritional modulation includes alteration of intermediates, precursors, catalyzers and regulators of many metabolic pathways. Therefore, nutritional interventions have the potential to simultaneously affect multiple targets that may be involved in the cancer cachexia process including energy intake, anorexia, inflammation and anabolic signaling (Figure 1). However, identification of an optimal nutritional intervention requires systematic experimental evaluation of the specific as well as combined effects of individual components.

Nutritional support in patients with cancer cachexia aims to counteract the negative energy balance as well as the net protein breakdown, without stimulating tumor growth or negatively influencing anti-tumor therapy. To establish a net positive protein balance, specific nutrients mitigating catabolic and stimulating anabolic signals should be considered. To create an anabolic environment, adequate caloric intake, and nutrient composition (e.g., quantity and quality of nutrients) is instrumental, because without sufficient nutrient availability, anabolic triggers will not lead to muscle maintenance or an increase in muscle mass. Several nutritional agents have been proposed to tackle cancer cachexia, however, clear evidence of their efficacy is limited. A better understanding of specific nutrients' contribution to muscle anabolism in these patients can lead to the development of specialized nutritional products focusing on halting muscle mass loss in cancer cachexia.

Experimental animal models are used extensively to study mechanisms underlying cancer cachexia and evaluate potential treatments. In this narrative review, based on a systematic evaluation of the current literature (Supplementary Table 1), we provide an overview of preclinical studies focused on nutritional interventions in cancer cachexia, and discuss the gaps and highlight opportunities in current experimental models.

## Single Nutrient Interventions

### Dietary Protein

Adequate supply of dietary protein is a prerequisite for maintenance or gain of skeletal muscle mass. A positive protein balance is required to increase skeletal muscle mass, and elevated plasma levels of essential amino acids from dietary protein are considered an effective anabolic stimulus (16). However, fundamental evidence on the sufficient and optimal quantity and quality of protein intake for treating low muscle mass is lacking. The ESPEN guidelines on nutrition in cancer patients suggest a protein intake in the range of 1.0–1.5 g/kg/day (17). Importantly, these guidelines are recommendations and evidence-based studies to support the optimal quantity are largely missing. Evidence concerning the quality of proteins, regarding the optimal amino acid composition and their digestibility (availability) in the context of cancer cachexia, is also lacking. In contrast to their suitability for defining the optimal quantity of protein resulting from translational challenges, experimental models of cancer cachexia can readily be deployed to study optimal amino acid composition and quality of proteins to prevent or treat cancer cachexia.

#### Branched-Chain Amino Acids

Branched-chain amino acids (BCAAs) have been hypothesized to exert a therapeutic effect in diseases accompanied by muscle wasting since they are integral components of skeletal muscle proteins and their critical role in stimulating protein synthesis (18). BCAAs may decrease proteolysis and increase protein synthesis in skeletal muscle, primarily through activation of the mTOR pathway and modulation of inflammation through glutamine production (19, 20). Of the BCAAs, leucine is most potent in stimulating muscle protein synthesis, whereas isoleucine and valine are less effective (20). In MAC16 tumor bearing mice, administration of leucine and valine significantly reversed the loss in body weight. Only leucine produced a significant recovery in muscle wet weight by increasing protein synthesis and decreasing degradation (20). In C26 tumor-bearing mice, leucine supplemented diet increased plasma amino acid concentration and counteracted muscle mass loss dose-dependently, while no effect of leucine-rich diet on body weight and/or anorexia was observed (21). In rats bearing the Walker 256 tumor, multiple studies have demonstrated that the loss of skeletal muscle mass induced by cancer cachexia was attenuated by leucine supplementation (22–26). Leucine supplementation did attenuate protein degradation, potentially through their modulatory effects on proteasome subunits; and improved protein synthesis, via activation of the mTOR pathway and downstream kinases. These actions were related to cachexia amelioration but did not increase survival time or reduce tumor growth (22, 23, 26, 27). Furthermore, a different cytokine profile was observed in tumor bearing rats fed a leucine-rich diet after 14 days. Both, the pro- inflammatory cytokines TNF-α, IL-6 and IFNγ and anti-inflammatory cytokines IL-10 and IL-4 were enhanced in the serum compared to tumor bearing control, indicating a cytokine modulatory effect (25). Altogether, these data suggest that leucine supplementation has a beneficial effect on experimental cancer cachexia. The BCAA leucine may attenuate muscle wasting by modulating protein synthesis and proteolysis.

#### β-Hydroxy-β-Methylbutyrate

β-hydroxy-β-methylbutyrate (HMB) is synthesized in the human body through metabolism of L-leucine (28). Under normal conditions about 5% of dietary leucine is converted into HMB (29, 30). This leucine metabolite is thought to modulate protein turnover, primarily by suppressing protein degradation (28). In mice bearing the MAC16 tumor, HMB supplementation attenuated the development of weight loss accompanied by a small reduction in tumor weight (31, 32). HMB caused a significant increase in the wet weight of soleus muscle and a reduction in protein degradation. Furthermore, Smith et al. showed that HMB was not only capable of attenuating protein degradation in skeletal muscle of cachectic mice but also stimulated protein synthesis (32). In rats bearing the Walker 256 tumor, HMB supplementation significantly decreased tumor weight. These rats maintained body weight, blood metabolic parameters (glucose, lactate, triacylglycerol, and cholesterol) and tissue glycogen levels comparable to non-tumor-bearing rats (33). Similar effects of HMB were obtained in rats bearing the Yoshida AH-130 tumor fed a 4% HMB-enriched diet (34). HMB supplementation led to a significant increase in body weight and a significantly attenuated gastrocnemius loss. Although, protein synthesis was not measured, HMB treatment increased phosphorylation of mTOR and p70S6k compared to both sham and tumor-bearing control, suggesting a direct modulatory effect on muscle protein anabolism (34). Given the beneficial effects of HMB on muscle protein turnover and the observed anti-tumor effect, HMB could be a useful supplement as part of the treatment of muscle wasting in cancer cachexia. However, further exploration of the efficacy of lower doses will be required, as the HMB doses used in these studies are supra-physiological and may not be feasible as intervention in a clinical setting.

#### Glutamine

Glutamine is the most abundant, non-essential, amino acid in the body that plays a role in a variety of physiological and biochemical processes (35). Glutamine is considered the main metabolic fuel for gastrointestinal enterocytes maintaining the normal integrity of the intestinal mucosa. Furthermore, glutamine plays an essential role as a precursor of peptide, protein, glucose and lipid synthesis (36, 37). Moreover, glutamine is one of the precursor amino acids of glutathione, which is a major antioxidant and a vital component of host defense (38). Although glutamine is the most abundant amino acid in the body, a marked glutamine depletion is observed in many patients with cancer (35). Consequently, glutamine might be useful to treat cancer cachexia. Supplementation with 2% L-glutamine showed to attenuate cancer-induced cachexia, indicated as preserved body weight loss and a lower cachexia index, in rats bearing the Walker 256 tumor (39–43). Furthermore, tumor growth was inhibited in tumor-bearing rats supplemented with L-glutamine (39–42). In cachectic rats, Walker 256 tumor growth caused considerable changes in small intestine metabolism (44). L-Glutamine supplementation restored the intestinal mucosa in the duodenum and jejunum by enhancing cell proliferation as well as increasing villous height, crypt depth, and total height of the intestinal wall (40). Furthermore, L-glutamine supplementation was associated with a significant elevation of glucose and insulin levels compared to control. The resulting hyperglycemia is probably attributable to the increased activity of gluconeogenic enzymes in the small intestine due to the increased availability of glutamine as a glucose precursor (41). All together, these studies suggest a beneficial effect of glutamine on cancer cachexia via enhancing intestinal health and energy metabolism.

#### Glycine

Glycine is a non-essential amino acid and is often considered biologically neutral. However, studies have shown that L-glycine has effective anti-inflammatory, immunomodulatory and cytoprotective properties (45). The underlying mechanisms responsible for the protective effects of glycine are not completely clear, but may include suppression of calcium signaling, inhibition of inflammatory cell activation and decreased formation of free radicals and other toxic compounds (45). In addition, dietary glycine was also reported to inhibit the growth of certain types of tumors, such as liver tumors (46) and melanoma tumors (47). Only one study investigated the effect of glycine treatment in an experimental model of cancer cachexia. In mice bearing the C26 tumor, glycine administration reduces tumor growth and attenuates cancer-induced cachexia (48). Glycine treatment partially prevented the tumor-induced loss in skeletal muscle mass and cross-sectional area. This protective effect of glycine was associated with preserved muscle function. In skeletal muscle, glycine normalized IL-6 and F4/80, a marker for macrophage in filtration, mRNA expression and reduced oxidative stress. In addition, glycine treatment attenuated Atrogin-1 and MuRF1 mRNA expression. In accordance, protein expression of the initiation factor eIF3-f, a major target of Atrogin-1 and an important regulator of protein synthesis, was preserved (48). This is the first and only study to demonstrate a beneficial effect of glycine on cancer-induced cachexia. Additional research is required to confirm these promising results and to further unravel the anti-tumor and anti-cachectic effects.

#### Arginine

Arginine is a conditionally essential amino acid, i.e., the body can synthesize sufficient amounts of arginine to meet physiological demands under well-nourished, healthy conditions (49). Arginine is involved in a number of biological processes including cell growth survival and protein synthesis. It is also a precursor in the biosynthesis of nitric oxide (50). Nitric oxide is a ubiquitous cellular messenger that stimulates the release of certain hormones, such as insulin and growth hormone, and is an important regulator of blood flow, tissue oxygenation, and immune function. In addition, arginine may enhance T cell natural killer cell activity, which inhibits tumor growth (51). Therefore, supplementation with arginine could be beneficial for patients with cancer cachexia through modulation of the endogenous anti-tumor response. However, up till now, there are no data in experimental cancer cachexia models published. This could be related to the concern that arginine, or its metabolites, interfere with metabolic pathways that can induce growth of some tumors (52, 53). These findings emphasize the importance that not all nutrients that envision positive effects on muscle anabolism are applicable in the treatment of cancer cachexia.

### Dietary Fat

Dietary fat is an important source of energy and contributes a significant caloric value to our diet. In Western diets, dietary fat may constitute 40–45% of the total caloric intake. Dietary fat is not just a source of energy, it also functions as structural component of cell membranes, carries fat soluble vitamins, plays an important role in signal transduction and is a precursor for inflammatory mediators (54).

#### High-Fat Ketogenic Diet

Currently, there is limited research available to substantiate an optimal energy percentage of dietary fat in cancer cachexia but the effect of ketogenic diets have been investigated in cancer. The ketogenic diet is a high-fat, low-carbohydrate diet designed to increase the blood concentration of free fatty acids and ketone bodies as alternative sources of energy to glucose (55). As tumor cells mostly rely on glucose as a substrate for anaerobic energy production, i.e., Warburg effect (56), ketogenic diets aim to reduce energy sources for the tumor, while providing free fatty acids and ketone bodies as an energy source for the muscle. Consequently, a high fat diet might be expected to prevent host catabolism during cachexia, mainly by tumor growth reduction. Only a few studies investigated the anti-cachectic effects of ketogenic diets in experimental models of cancer cachexia. In mice bearing the MAC16 tumor, Tisdale et al. showed that the cachectic phenotype can be partly reversed by a ketogenic diet [80% of calories supplied as medium chain triglycerides (MCT)], causing reduced tumor growth and an inhibition of body weight loss (57). Body composition analysis showed retention of both fat and fat-free carcass mass in animals fed high levels of MCT. In another study, an 80% MCT-based high-fat diet reduced both tumor weight and host weight loss and restored both nitrogen balance and urea excretion to that of non-tumor-bearing mice (58). Furthermore, amino acid concentrations in plasma were also restored to control levels, suggesting that excessive nitrogen catabolism during cachexia can be prevented. More recently, the effect of an infant formula with a ketogenic composition used to treat patients with refractory epilepsy was investigated on cancer cachexia in C26 tumor-bearing mice (59). Mice receiving the ketogenic formula showed reduced body weight loss and muscle mass loss. Tumor growth and plasma IL-6 levels were significantly decreased in mice receiving the ketogenic formula compared to tumor-bearing control. It seems that the ketogenic diet inhibits tumor growth and thereby prevents host catabolism. Collectively, these studies suggest that the ketogenic diet with adequate amounts of proteins has beneficial effects on the development of cancer cachexia. However, there is some debate concerning the use of ketogenic diets and the development of dyslipidemia. Some (60, 61) but not all (62) studies indicate that a ketogenic diet produce significant increases in the plasma levels of total cholesterol and triglycerides. Elevated levels of plasma triglycerides and cholesterol are often detected in patients suffering from cancer cachexia (63), as a result of increased lipolysis (64, 65). This tumor induced dyslipidemia, in turn causes lipotoxic effects in other tissues including skeletal muscle (65, 66). Considering the potential impact of dyslipidemia to cancer cachexia, this potential effect should be further investigated to probe the feasibility of the ketogenic diets in patients with cancer cachexia.

#### Polyunsaturated Fatty Acids

Dietary long chain polyunsaturated fatty acids (lcPUFAs) have an effect on diverse physiological processes affecting normal health and chronic diseases (67–69). The n-3 and n-6 lcPUFA families are derived from the desaturation and elongation of the essential lcPUFAs α-linolenic and linoleic acids that are ingested as components of food. The principal members of the n-3 lcPUFA family are eicosapentaenoic acid (EPA) and docosahexaenoic acid (DHA), whereas arachidonic acid is the main derivate of the n-6 lcPUFA family. It is known that eicosanoids derived from n-6 lcPUFAs have pro-inflammatory and immune-active functions, whereas n-3 lcPUFA-derived eicosanoids have anti-inflammatory properties, attributable to their ability to inhibit the formation of n-6 lcPUFA-derived eicosanoids.

Diet supplementation with fish oil, which is rich in n-3 lcPUFAs EPA and DHA, has been investigated to preserve skeletal muscle mass in various experimental animal models of cancer cachexia. Most of these studies show that fish oil is an effective nutritional intervention to induce body weight gain, reduce tumor growth rate and reverse food intake. In 1990, Tisdale et al. showed for the first time that a diet enriched in fish oil reduced both tumor growth and weight loss produced by the MAC16 adenocarcinoma (70). Reversal of host body weight loss was associated with an increase in total body fat and muscle mass. Although the amount of fish oil required for optimal activity was high (50% of total calories), no toxicities were observed (70). Comparable results were found in Walker 256 tumor-bearing rats receiving an n-3 fish oil diet. Tumor weight gain in fish oil fed rats was reduced and these animals gained body weight and maintained blood metabolic parameters (glucose, lactate, triacylglycerol and cholesterol) similar to non-tumor-bearing rats (71). Fish oil supplementation via oral gavage reduced body weight loss and tumor weight gain to a similar extent (72). Finally, some studies investigated the effect of lifelong supplementation of the diet with fish oil on cancer cachexia (73, 74). In these studies, the diet of pregnant and lactating rats and subsequent male offspring was supplemented with fish oil. Lifelong supplementation of the diet with fish oil significantly decreased tumor growth, increased survival, reversed food intake and prevented body weight loss. Furthermore, fish oil supplementation partly preserved blood metabolic parameters in tumor-bearing rats compared to control (73, 74). However, it is difficult to predict if the effects found in these lifelong supplementation studies can be translated into a treatment regimen relevant to cancer cachexia.

While most studies reported clinically and statistically significant effects of fish oil supplementation on preventing body weight loss, Dumas et al. have found no effect (75). In rats with peritoneal carcinosis, fish oil-enriched diet delayed the occurrence of anorexia compared to the control diet. Furthermore, ascites production was lower in fish oil treated rats. However, no difference in body weight gain, fat mass and muscle mass was reported (75). The discrepancies could be due to differences in the design of the study. In the aforementioned studies, in both the treatment arm and control arm relative weight gain of animals and tissues were assessed at the same time point, whereas Dumas et al. assessed the final measurements when anorexia was evident in all animals, ensuing different time points per group.

Besides fish oil, shark liver oil is a common dietary supplement rich in n-3 lcPUFAs. Prophylactic supplementation with shark liver oil promoted gain in body weight, reduction of tumor weight and maintained blood metabolic parameters in Walker 256 tumor bearing rats (76, 77). However, shark liver oil supplementation showed less potent effects compared to fish oil (77).

The anti-cachectic and anti-tumor effect of purified EPA has also been investigated in various experimental animal models. In mice bearing the cachexia-inducing MAC16 adenocarcinoma, EPA was found to stabilize weight loss, delay growth of the tumor, and increase the overall survival (78–80). Such anti-cachectic effect was not achieved by the use of DHA or linoleic acid alone (79). Supplementation with EPA inhibited tumor-induced lipolysis and reduced protein degradation without an effect on protein synthesis (78, 81, 82). In mice bearing the cachexia-inducing S180 ascites tumor, oral administration of EPA prevented body weight loss by preserving the white adipose tissue mass (83). Furthermore, EPA administration suppressed plasma levels of pro-inflammatory cytokines such as TNF-α and IL-6. Lastly, EPA treatment also preserved several key lipolytic factors and raised the mRNA levels of some adipogenic factors in the white adipose tissue (83). A higher body weight gain in response to EPA supplementation has also been reported in Apc^(min/+)^ mice (84), rats implanted with MCA sarcoma (85), and rats treated with MAT-LyLu prostate tumor cells (86).

Taken together, these studies suggest that n-3 lcPUFAs, in particular EPA, are effective in preventing body weight loss and tumor growth in experimental animal models of cancer cachexia. However, little is known on the underlying mechanisms of n-3 lcPUFAs/EPA in cancer cachexia. The aforementioned data suggest that n-3 lcPUFAs/EPA is able to suppress lipolysis, probably by downregulating lipid mobilizing factors (87). Whether the anti-lipolytic activity is a direct effect of n-3 lcPUFAs/EPA or related to its anti-inflammatory properties through inhibiting pro-inflammatory cytokines, such as TNF- α and IL-6, needs to be further elucidated.

#### Conjugated Linoleic Acid

Conjugated linoleic acid (CLA) is a group of at least 28 isomers of linoleic acid found mostly in red meat and dairy products derived from ruminants (88). CLA is marketed as a weight-loss supplement to reduce body fat and promote lean muscle growth (e.g., Tonalin®). Experimental studies have shown that CLA has anti-carcinogenic effects (89, 90). Furthermore, CLA was protective against TNF- α and LPS induced muscle wasting (91, 92). However, data on the effect of dietary supplementation of CLA in the treatment of cancer cachexia is contradictory. In mice bearing the C26 tumor, Graves et al. showed that a diet containing 0.5% CLA preserved skeletal muscle mass and reduced TNF receptor type 1 levels in muscle homogenates (93). In mice bearing the LLC tumor, the same diet reduced skeletal muscle wasting, but had no effect on skeletal muscle levels of TNF receptor type 1 (94). In mice bearing the B16 melanoma, 0.5% CLA did not affect skeletal muscle mass or TNF receptor type 1 levels in skeletal muscle (94). The notion that CLA has beneficial effects in cancer cachexia was also not supported by Tian et al. (95). In this study, a 1% CLA enriched diet did not affect skeletal muscle mass and adipose tissue mass. CLA supplementation did not inhibit the induction of proteolytic markers. Instead, MuRF1 expression was significantly higher in C26 tumor-bearing mice receiving a diet containing 1% CLA. In skeletal muscle, CLA enhanced tumor- induced gene expression of inflammatory markers TNF- α, IL-6 receptor and F4/80. Moreover, in epididymal adipose tissue, tumor driven lipolysis was aggravated by CLA supplementation (95). More recently, in rats bearing the Walker 256 tumor, CLA treatment aggravated cachexia symptoms, including increased inflammatory status, steatosis and hyperlipidemia (96). Collectively, these results do not provide strong support for CLA in the treatment of cancer cachexia.

### Other Nutrients

#### Carnitine

Carnitine is a trimethylamine, which is synthesized in the liver and kidney via the conversion of two essential amino acids, lysine and methionine. Carnitine plays a major role in the import of long chain fatty acids from the cytosol into the mitochondrial matrix for subsequent β-oxidation (97, 98). Inefficiency of this bioenergetics process results in increased oxidative stress, contributing to the development of metabolic abnormalities and the release of pro-inflammatory cytokines. Consequently, supplementation of carnitine to enhance mitochondrial β-oxidation may attenuate oxidative stress and inflammation, resulting in beneficial clinical outcomes. Carnitine supplementation has been studied in various experimental models of cancer cachexia. In rats bearing the Yoshida AH-130 tumor, L-carnitine treatment resulted in greater food intake (99, 100). Protein levels of carnitine palmitoyl transferase-1 (CPT-1) enzyme, a marker of carnitine effects, were higher in the muscles of tumor-bearing rats treated with L-carnitine compared with the non-treated cachectic animals (100). This was accompanied by an inhibition of tumor-induced muscle wasting (99, 100) and an increase in physical activity (99). Moreover, L-carnitine treatment resulted in a down-regulation of atrogin-1 and MuRF1 and a decrease in the proteasome activity in gastrocnemius muscle (99, 100). In addition, the pro-apoptotic marker caspase-3 in skeletal muscle tended to be decreased in muscle of tumor-bearing rats treated with L-carnitine (99). In mice bearing the C26 tumor, L-carnitine significantly increased food intake, muscle mass and epididymis fat weight through the upregulation of CPT (101). In addition, the increased CPT activity was associated with reduced plasma levels of IL-6 and TNF-α (101). In cachectic rats bearing the Walker 256 tumor, CPT activity was reduced and liver and plasma triacylglycerol content was increased. L-Carnitine treatment restored these measures back to control values, showing that L-carnitine preserves hepatic lipid metabolism in experimental cancer cachexia (102). Current evidence suggests that carnitine might help to ameliorate muscle wasting in cancer, although more molecular studies investigating the exact working mechanism are needed.

#### Creatine

Creatine is a non-protein amino acid that can be endogenously synthesized in the liver, kidney and pancreas and is mainly stored and utilized in the skeletal muscle. Creatine can be phosphorylated by creatine kinase to form phosphocreatine, which plays an important bioenergetics role by providing rapid energy during muscle contraction, where phosphocreatine donates a phosphate group to adenosine diphosphate to resynthesize adenosine triphosphate (103, 104). Given that oral creatine supplementation augments its intramuscular content and has the capacity to effectively enhance muscle strength and lean body mass (105–107), makes it an interesting supplement to treat muscle-wasting diseases. Indeed, creatine supplementation has been successfully used as an adjuvant treatment in numerous myopathies (108–110). Furthermore, creatine supplementation has shown antioxidant capacities as well as effectiveness to counteract pro-inflammatory cytokines (111, 112). Despite the promising results, only a few studies have investigated the effects of creatine supplementation in experimental cancer cachexia. In rats bearing the Walker 256 tumor, creatine supplementation attenuated body weight loss and tumor growth was decreased (113–115). Cancer-induced skeletal muscle atrophy was attenuated by creatine, as evidenced by the increase in muscle fiber cross-sectional area. Creatine also prevented cancer-induced increase in Atrogin-1 and MuRF1 protein levels. Furthermore, creatine supplementation prevented the increase in plasma TNF-α and IL-6 (113, 114), while it increased plasma IL-10 (114). However, mean survival time was not different compared to tumor-bearing control (113, 115). Given the beneficial effects of creatine supplementation on skeletal muscle mass maintenance in experimental cancer cachexia and the promising results in other muscle wasting diseases, creatine could be a useful supplement to treat muscle wasting in cancer cachexia and should be the objective for future studies. It should be pointed out however that creatine supplementation may increase urinary creatinine levels, which complicates interpretation of the latter as a marker of renal dysfunction in patients with a history or risk of renal disease.

#### Flavonoids

Flavonoids are a large group of polyphenolic compounds and are ubiquitously expressed in plants. Fruits and vegetables are the main dietary sources of flavonoids for humans, along with tea and wine. As a dietary component, flavonoids are thought to have health-promoting properties due to their antioxidant, hepatoprotective, anti-inflammatory and anti-carcinogenic properties (116). Despite several known effects of flavonoids on health and disease, research into the effects of flavonoids on cachexia prevention has been limited to date. In Apc^(min/+)^ mice, quercetin supplementation attenuated the progression of cancer cachexia (117). Quercetin significantly attenuated body weight loss, but did not affect the loss of epididymal fat in Apc^(min/+)^ mice. After 3 weeks of supplementation, the loss of muscle mass and grip strength shown in Apc^(min/+)^ mice was significantly attenuated by quercetin. Furthermore, increased plasma IL-6 levels were completely mitigated by quercetin in this model of intestinal cancer. In this study, no effect of quercetin supplementation on tumor number was observed, while a reduction of the tumor weight was found in rats bearing the Walker 256 tumor (118). Epigallocatechin-3-gallate (EGCG), the principal polyphenolic component in green tea, effectively attenuates skeletal muscle atrophy in mice bearing the Lewis lung carcinoma (LLC) tumor (119). EGCG supplementation inhibited tumor growth and the loss of body weight was significantly slowed down. Furthermore, it was shown that EGCG positively regulates the expression of muscle-specific ubiquitin ligase genes encoding MuRF-1 and Atrogin-1 (119). Another study examined the effect of isoflavones derived from soy extracts on muscle atrophy in LLC-bearing mice (120). The isoflavone diet attenuated the tumor-induced loss in wet weight and myofiber size of the gastrocnemius muscle. Moreover, the increased expression of MuRF-1 and Atrogin-1 was significantly suppressed by the supplementation of isoflavones. In parallel, the isoflavone diet significantly inhibited the phosphorylation of ERK in skeletal muscle of tumor-bearing mice. No effect of dietary isoflavones on tumor mass or pro-inflammatory cytokines IL-6 and TNF-α were observed (120). Morin, another flavonoid, was able to suppress tumor-induced skeletal muscle wasting in LLC-bearing mice (121). Dietary morin prevented the reduction of muscle wet weight and myofiber size. Moreover, the tumor mass in mice fed the morin diet was significant lower compared to mice fed the normal diet. It was suggested that morin indirectly prevents muscle atrophy by suppressing tumor growth via targeting ribosomal protein S10. The anti-proliferative effect of morin had a cell specific action that was only apparent in tumor cells, but not in muscle cells (121). Because of the variation in flavonoids and limited number of studies, there is not enough evidence to recommend flavonoids as a potential supplement to treat muscle wasting in cancer. However, the available data merits further investigation of their potential to modulate cancer cachexia.

#### Resveratrol

Resveratrol (3,5,4′-trihydroxystilbene) is a phytoalexin, a class of compounds produced by many plants when they are infected or physically harmed (122); it has been reported to have anti-tumor effects in rats (123). Research on the potential positive effects of resveratrol in cancer cachexia is limited and contradictory. In mice bearing the MAC16 tumor with established weight loss, resveratrol partly blocked weight loss by interfering with NF-κB activity in skeletal muscle and this was accompanied by inhibition of tumor growth (124). Another study demonstrated that resveratrol inhibited skeletal muscle atrophy induced by the C26 adenocarcinoma tumor through its inhibition of NF-κB activity in skeletal muscle (125), without affecting tumor growth. In contrast to these studies, administration of resveratrol did not attenuate skeletal muscle mass loss or body weight loss in both rats bearing the Yoshida AH-130 tumor and mice bearing the LLC (126). In fact, in rats, administration of resveratrol exaggerated cancer cachexia (126). On the contrary, resveratrol was able to decrease proteolysis *in vitro* (126). After absorption, resveratrol undergoes rapid and extensive metabolism leading to low bioavailability (127, 128). With respect to the poor bioavailability it might be not surprising that resveratrol did not prevent muscle wasting, as the likelihood to reach effective levels in plasma or muscle is very low. Taking these aforementioned studies into consideration, resveratrol is not likely able to ameliorate tumor induced cachexia.

#### Prebiotic Non-digestible Oligosaccharides

A prebiotic is “a selectively fermented ingredient that allows specific changes, both in the composition and/or activity in the gastrointestinal microflora that confers benefits upon host well-being and health” (129). Prebiotic oligosaccharides such as galacto-oligosaccharides (GOS) and fructo-oligosaccharides (FOS) have shown to have immune modulating activities, observed in several animal experiments, and clinical trials (130–132). These oligosaccharides have been associated with improved gut barrier function (133). It stimulates bifidobacteria, lactobacilli and other healthy bacteria, while it reduces harmful bacteria in the gut. Moreover, these oligosaccharides may block or activate specific receptors on immune cells leading to improved immune responses. In addition, oligosaccharides are fermented by colonic bacteria into short-chain fatty acids (133). Short-chain fatty acids have been shown to exert anti-carcinogenic as well as anti-inflammatory properties (134). Although, the exact role of the microbiome in cancer cachexia is not clear yet, animal studies suggest that the composition of the microbiota and intestinal barrier function is affected by the presence of a tumor and the development of cancer cachexia (135). These findings support the rational to target the gut microbiota in cancer cachexia using prebiotic non-digestible oligosaccharides. The therapeutic potential of prebiotic non-digestible oligosaccharides in cancer cachexia have been barely explored. In mice bearing the C26 tumor, diet supplemented with GOS and FOS (weight ratio 9:1) significantly reduced skeletal muscle mass loss (136). Similar results were obtained when the FOS was replaced by additional GOS (136). Administration of a prebiotic candidate, pectic oligosaccharide (POS), to leukemic mice mitigated the cachectic phenotype, by delaying anorexia and preserving fat mass (137). In addition, POS increased the total number of bacteria and induced a drastic change in microbial diversity and populations. No effect on inflammation was observed (137). Due to limited research, the impact of prebiotic non-digestible oligosaccharides on cancer cachexia is currently unclear and would warrants further investigation.

## Multinutrient Interventions

As discussed in section Single nutrient interventions, numerous nutrients led to positive results in experimental models of cancer cachexia, targeting different aspects of the wasting syndrome. Combining such high quality nutrients in a “multinutrient approach” to treat cachexia, is expected to have a superior impact compared to single nutrients. A multinutrient approach is aimed at targeting specific factors involved in cancer cachexia, e.g., anorexia, altered fat, and protein metabolism, and systemic inflammation, but also the replenishment of nutrient deficiencies.

Several of these multi-nutrient approaches have been studied in experimental models of cancer cachexia. In mice bearing the MAC16 tumor, Smith et al. investigated the effect EPA combined with protein (casein), amino acid mixture (leucine, arginine and methionine), and carbohydrate on protein synthesis and degradation in gastrocnemius muscle (82). Treatment with only EPA significantly reduced protein degradation, but had no effect on protein synthesis. Combination of EPA with casein tended to increase protein synthesis. However, when combined with the amino acid mixture, protein synthesis almost doubled. The addition of carbohydrate to stimulate insulin release had no additional effect (82). In another study, Van Norren et al. examined the effect of dietary supplementation with a specific combination of high protein (100% casein), leucine and fish oil (EPA and DHA in a ratio of 2.2:1) on body composition in mice bearing the C26 tumor (138). The multinutrient intervention targeting catabolism, anabolism and essential amino acid supply significantly reduced loss of carcass, muscle and fat mass. Addition of the single nutrients to the diets resulted in no or marginal effects (138). In a second experiment, the effect of a more humanized diet on weight loss, muscle function and physical activity was studied, referred to as Specific Nutritional Composition, containing high protein (68% casein and 32% whey), leucine, fish oil (EPA and DHA in a ratio of 2.2:1), and the oligosaccharides GOS and FOS. The specific nutritional composition diet significantly reduced loss of body, carcass, muscle and fat mass. Furthermore, tumor weight was significantly lower compared to tumor-bearing control. Muscle performance was improved and total daily activity was normalized after intervention with the specific nutritional composition diet (138, 139). The specific nutritional composition diet also showed beneficial immune modulatory effects. The nutritional combination significantly improved the Th1 immunity and plasma levels of IL-6, TNF-α and PGE_2_ were significantly reduced (139). Furthermore, this combination attenuated weight loss and inflammatory markers, and reduced pathogen levels and bacterial translocation in a chemotherapy model (140). In mice bearing the B16 melanoma, the effect of a multi-nutrient intervention with BCAAs, citric acid, L-carnitine, coenzyme Q10 and various vitamins and minerals was investigated on the development of cancer cachexia (141). The mice receiving the intervention diet showed a higher cumulative food intake compared to tumor-bearing control. In addition, tumor weight was significantly lower and lung metastasis of the B16 melanoma cells were absent in the intervention group. However, attenuation of muscle tissue loss was only observed for the suprahyoid muscles in the neck but not for gastrocnemius or soleus muscles in the lower leg (141). More recently, the effect of total nutrition formula on LLC-bearing mice was investigated (142). Total nutritional formula is an energy- and protein-dense oral nutritional supplement fortified with micronutrients such as vitamin D, vitamin A, coenzyme Q10 and selenium. Daily oral supplementation with total nutritional formula significantly suppressed tumor growth, while body weight loss and gastrocnemius muscle mass loss were significantly attenuated. Furthermore, the plasma levels of TNF-α and IL-6 were significantly decreased in LLC-bearing mice supplemented with total nutritional formula (142).

Clearly, the few studies in which the combination of nutrients was systemically compared to single nutrients alone strongly support the concept of a multinutrient approach. More experimental dietary intervention studies should focus on multi-nutrient interventions targeting multiple aspects of the disease. Furthermore, future studies should define optimal selection and balance of nutrients that work in concert to produce a health benefit that is greater than the sum of the single nutrients.

## Nutritional Interventions: Gaps and Opportunities

All of the nutritional intervention studies included in this review describe effects on phenotypic measures of cachexia, such as body weight loss and/or muscle mass loss. However, only few of the studies contribute to understanding the underlying mechanisms by which beneficial effects of nutritional interventions are mediated. Of all reviewed nutrients, only L-leucine and its metabolite HMB have been shown to attenuate tumor-induced muscle wasting by (directly) modulating muscle protein synthesis and proteolysis. For many other nutrients, the anti-cachectic effect is frequently associated with reduced tumor growth, modulation of inflammatory signaling or other extra-muscular alterations that may contribute to cachexia. In these studies, it is difficult to discriminate whether these nutrients act directly on skeletal muscle metabolism, or impact indirectly on cachexia. A better understanding of the beneficial actions of nutritional interventions will not only aid their clinical implementation, but also the systematic evaluation of additive or synergistic effects of rationally selected nutritional combinations with distinct or complementary actions.

### Standardization in Experimental Design

Given the heterogeneity of cancer-induced cachexia, the results obtained in the reviewed studies must be considered in the context of the specific models that were used, and variations in dietary regiments that were applied. Although these variations may cause discrepancies between results across different animal models, and laboratory groups, preclinical studies *per se* have provided advantages and results that merit their application in cachexia research. The experimental models of cancer cachexia allow analysis of different tissues affected by cancer cachexia, and provide valuable insights in the underlying mechanisms of tested interventions. In addition, safety and efficacy parameters that are relevant and translatable to clinical practice are defined in experimental models, including identification of potentially harmful interactions with other treatment modalities. Nevertheless, various aspects in the experimental design would benefit from standardization. When evaluating the potential beneficial effects of the nutritional interventions in experimental cancer cachexia, a wide variation in timing, duration, dosing and route of supplementation was observed, as well as in animal models used (Supplementary Table 1).

#### Intervention as Source of Variation

Dietary intake, composition and dose is an often overlooked source of variation in experimental animal studies of cancer cachexia (143). The majority of the studies reviewed provide diets *ad libitum*, while only a few studies control daily food intake. However, it should be kept in mind that anorexia is an important contributing factor to cancer-induced muscle wasting and that dietary intake is closely related to cachexia-related outcome measures. This stresses the importance of dietary standardization, recording of food intake or pair feeding the control group, to correlate the amount of nutrient intake with outcome measures. Details on dietary intake are often provided, however, the exact composition of the experimental diets is often not described. Many of the studies reviewed used standard laboratory chow. Standard laboratory chow diets do not have a standardized macronutrient composition and often vary from batch to batch (144). Furthermore, the ingredients of laboratory chow are rarely defined, therefore it might contain unknown nutrients that may impact study endpoints. The use of purified or semi-purified diets should be the new standard. In comparison to standard laboratory chow, the ingredients included in purified and semi-purified diets are open formulas and well-characterized (145). In addition, the formulation of the semi-purified diet can be altered by the researcher according to the research objective. Besides the choice of the background diet, the control diet and intervention diet should be matched for calories and nitrogen content. The proportions of the nutrients in relation to human intake should also be considered, improving the translatability to the clinic. Furthermore, the dose of the supplemented nutrients is a point of attention. Some of the intervention studies use supraphysiological doses that may not be feasible for a nutritional intervention. For example, a diet with 4% HMB is rather unbalanced considering that normal rodent chow contains only 12.5% protein.

The timing and duration of supplementation as well as the route of administration vary between studies and is another source of variation. The majority of the studies started supplementation before or with tumor inoculation, while only a few initiate supplementation when animals show evidence of cachexia. In both study designs, the metabolic state of the animals at the start of the intervention is different, each of which may require a different formulation of an effective nutritional intervention. Besides, nutrients might have a fundamental different action in such models, for example by affecting tumor take or growth. In addition to the timing, the duration of the intervention studies varies substantially, ranging from 24 h to 34 days after tumor inoculation. This suggests that the cachectic state at the end of the study differs between these studies, making it difficult to compare phenotypical and biochemical data between studies.

In addition, different routes of administration were applied in the reviewed experimental intervention studies. Only a few administrated the intervention via intra-peritoneal injection, subcutaneous injection or drinking water. A significant number of studies supplemented the nutrients via oral gavage. Similar to the injections, the advantage of administration via oral gavage is that an exact amount of the nutrient is delivered irrespective of the food intake, which minimizes variation between animals. However, by administration via oral gavage the nutrient appears as a bolus and is not part of the food matrix. Furthermore, administration via oral gavage may have impact on the food intake and the intervention diet is often not isonitrogenous and isocaloric to the control diet. In the majority of the studies the nutrients are administered via the diet, either by modification of semi-purified diets, or by incorporation into the standard laboratory chow. This allows to explore the full metabolic potential of nutritional interventions, as it takes its processing through the complete digestive tract into consideration. Moreover, from a translational perspective this may be the preferred route of administration, as its clinical application will be accordingly.

Combined, systematic attention to the aspects discussed above will contribute to further standardization in experimental design and reporting of experimental details. In turn, this will greatly stimulate the speed to explore promising leads and turn these into reproducible preclinical intervention diets, with robust translational potential in cancer cachexia.

#### Heterogeneity Between Animal Models of Cancer Cachexia

Numerous well-established animal models of cancer cachexia are used (Table 1). However, there is substantial variability between them in terms of cell type, site of inoculation/implantation, tumor growth (speed and size), development of metastasis, the overall dynamics of the wasting process, and putative underlying mechanisms (e.g., anorexia, inflammation) (146). This makes it difficult to compare results across animal models. Furthermore, these animal models often do not recapitulate all major clinical characteristics present in cancer cachexia, which may complicate direct translation of the findings to the clinic. For example, the kinetics by which cachexia develops in these models differs from that in patients. The effectiveness of some treatments may be of transient nature and lost in patients with chronic cachexia, while treatments that do not prove effective in acute models of cachexia might be useful for treating chronic cachexia. Furthermore, the majority of the animal models have ectopically growing tumors. Although, these animal models have been useful in investigating the underlying mechanisms of cancer cachexia, animal models of cancer cachexia with orthotopically growing tumors may more closely represent clinical cancer cachexia. Orthotopic tumor growth provides tumor cells its original stroma and microenvironment, both of these elements may determine the etiology of cachexia and affect the outcome of interventions. Indeed, recently, it has been demonstrated for a murine model of pancreatic cancer cachexia that orthotopic implantation of tumor cells on the location of interest much better recapitulates the clinical characteristics of cancer cachexia compared to subcutaneous tumor models (147). The growth monitoring capabilities for palpable, subcutaneously grown tumors is no longer a rationale for avoiding the use of orthotopic models, as recent studies have demonstrated the routine deployment of non-invasive imaging in various models, including orthotopically grown lung and brain tumors, to follow up tumor growth (148–150). Moreover, micro-CT imaging-based automated 3D contouring algorithms have been developed to simultaneously determine muscle mass changes in a model of orthotopic lung cancer cachexia (151). Finally, orthotopic tumor models provide an opportunity to study the interaction between conventional cancer therapies (e.g., chemo-, immune-, and radiation therapy) and adjuvant interventions targeting cachexia. Taken together, the development of more humanized models to study the effectiveness of interventions to treat cancer cachexia may lead to a more rapid translation into clinical trials.

**Table 1:** Heterogeneity between well-characterized animal models of cancer cachexia.

**Model**	**Tumor origin**	**Host**	**Tumor injection site**	**Experimental period (days post tumor injection)**	**Anorexia**	**Inflammation**	**Metastasis**
**MOUSE MODELS**							
C26	Colon	CD2F1 and BALB/c	s.c. or i.m.	11–21	No/Yes depending on experimental design	Yes (IL-6)	No
LLC	Lung	C57Bl/6	s.c. or i.m.	15–34	Yes	Yes (TNF-α, IL-6, IFN-γ)	Yes
Apc^(min/+)^	Colon	C57Bl/6	Genetic		No	Yes (IL-6)	No
MAC16	Colon	NMRI mice	s.c.	1–20	No	Yes (TNF-α, IL-6, IL-1)	No
B16	Melanoma	C57Bl/6	s.c.	16	Yes	Yes (TNF-α, IL-6)	No
**RAT MODELS**							
Walker 256	Mammary gland	Wistar rats	s.c.	7–33	Yes	Yes (TNF-α, IL-6, PGE_2_, Walker factor)	No
Yoshida AH-130	Ascites hepatoma	Wistar rats	i.p.	7–24	Yes	Yes (TNF-α, PGE_2_)	No
MAT-LyLu	Prostate	Copenhagen Fisher rats	s.c.	14	No	Not reported	Yes

*s.c., subcutaneous; i.m., intramuscular, and i.p., intraperitoneal*.

*Tumor volume varies significantly between models. This parameter might affect the translatability to the clinical situation. However, the data provided in the reviewed papers did not provide sufficient information to summarize relative or absolute tumor volume*.

### Combining Nutrition With Anti-cancer Therapy and Cachexia Interventions

Although not in the scope of this review, in addition to tumor activity and host responses that may drive cachexia, there is increasing clinical evidence that treatments directed at the tumor may actually contribute to the development and progression of cachexia (152, 153). Studies in healthy mice and rats have also demonstrated that some chemotherapeutic agents induce muscle wasting (154, 155). Recently, it was for the first time demonstrated that distinct metabolic derangements are present in cancer-induced and chemotherapy-induced cachexia (156). This implies that different therapeutic strategies to treat cachexia may be required. A better understanding of the tumor-, host-, and treatment-induced drivers of cachexia in combination with in-depth knowledge on the mechanism of nutritional interventions is crucial to develop effective therapeutic interventions for cancer-induced and treatment-induced cachexia. Moreover, as in practice nutritional interventions will be used in combination with anti-cancer therapies such as chemo-, radiation- and immunotherapy, it is necessary to investigate if such combinations are safe, and whether they modulate the therapeutic effectiveness. Some nutrients, such as lcPUFAs, may improve the adherence to and efficacy of chemotherapy (157), while others, including carnitine and glutamine are suggested to reduce the toxicity of anti-cancer therapy in cachectic patients (158).

Considering cancer cachexia is a multifactorial syndrome, it is expected that a multimodal approach targeting multiple aberrant pathophysiologic pathways simultaneously will be most efficient. In addition to optimized nutritional care, other therapeutic strategies, including exercise training and pharmacological interventions, could effectively contribute to attenuate cancer related muscle wasting. Although not all treatment modalities can be modeled in pre-clinical experimental set ups, efficacy, synergy and relevance of some combined treatment modalities have been evaluated. In particular, exercise training has some experimental evidence of benefit to mitigate skeletal muscle loss and therefore should be seen as potential tool to maximize outcomes and quality of life of patients with cancer cachexia (159–162). Exercise training stimulates the increase of muscle mass and strength and might improve cancer-associated skeletal muscle wasting by stimulating anabolic pathways as well as by down-regulating the activity of pro-inflammatory cytokines (163). In addition, exercise training has a beneficial effect on counteracting fatigue, which is one of the most severe symptoms in cancer patients (164). Combining exercise training and nutritional intervention was shown to be more effective than either one alone, highlighting the potential of multimodal interventions with a nutritional component (159, 161, 162). Besides exercise training, pharmacological agents such as appetite stimulants, anabolic steroids, and non-steroidal anti-inflammatory drugs could effectively contribute to the treatment of cachexia (165). Carefully designed combination therapy may maximize the impact on treating muscle wasting while minimizing the chance for drug toxicities. Various combinations of single nutrients with exercise or pharmacological agents have been studied in experimental cancer cachexia, with some success. However, the number of studies is limited and therefore more research is needed to find the optimal combination. In future studies, attention should be paid on the interaction between the different components of the multimodal approach. It is important that the combination is safe and contributes to the total therapy effectiveness.

## Conclusion

Targeted nutrition is pivotal in preserving muscle mass in cancer cachexia. A large number of studies demonstrated beneficial effects of nutritional interventions on muscle wasting. However, evidenced-based studies that indicate the optimal quantity and quality of the nutrients are often missing. Furthermore, the combination of multiple nutrients is expected to have a superior impact compared to single nutrients alone. More studies investigating the optimal quantity, quality and combination of nutrients are needed. Rationally-designed nutrition intervention studies, performed in well-characterized experimental models are an essential approach in the design of multimodal therapies targeting cancer cachexia.

## Author Contributions

WW has drafted the manuscript. WW, AS, JT, AH, and RL edited and revised the manuscript. All authors listed have approved it for publication.

## Conflict of Interest

AH was employed by Nutricia Research. The remaining authors declare that the research was conducted in the absence of any commercial or financial relationships that could be construed as a potential conflict of interest.
