# Supplementary material for: Nutritional Interventions in Cancer Cachexia: Evidence and Perspectives From Experimental Models
Source: Front Nutr. 2020 Dec 22;7:601329. doi: 10.3389/fnut.2020.601329 (PMC7783418; doi:10.3389/fnut.2020.601329)
Supplement: Supplementary file 1 [file Table_1.docx]

| **Supplemental Table 1:** Overview of reviewed studies showing the variation in timing, duration, dosing and route of supplementation, as well as the differences in animal models used in the experimental models. | | | | | | | | | | |
| --- | --- | --- | --- | --- | --- | --- | --- | --- | --- | --- |
| **Reference** | **Animal + tumor model** | **Experimental**  **groups** | | | **Timing (start intervention)** | **Duration** | **Dose/route of administration** | **Background diet** | **Control diet Isocaloric/isonitrogenous** | **Diet control** |
| ***Branched-chain amino acids*** | | | | | | | | | | |
| Eley et al., 2007 | NMRI mice – MAC16 colon | TB  TBN | | | When animals had lost approx. 5% of their starting body weight. | Animals were euthanized when the body weight loss reached 20% of their starting weight. (4-5 days) | **(1) Control group:** PBS, daily by **oral gavage**;  **(2) Leucine group:** 1 g of leucine per kg bodyweight, daily by **oral gavage.** | Rat and mouse breeding diet (Special Diet Services) | Detail on energy and nitrogen correction not specified. | Ad libitum |
| Peters et al., 2011 | CD2F1 (BALB/c x DBA/2) mice – Colon 26 | C  TB  TBN | | | With tumor injection | 21 days | **(1) Low leucine** (1g/kg food)- containing 9,6% Leu per g protein;  **(2) High leucine** (8g/kg food) - containing 14,8% Leu per g protein. | AIN-93M (Research Diet Services) | Detail on energy and nitrogen correction not specified. | Ad libitum |
| Ventrucci et al., 2004 | Wistar rats – Walker 256 (pregnant) | C  TB  P  CN  TBN  PN | | | immediately after the confirmation of pregnancy | 21 days | **(1) Control** AIN-93G modified diet containing 18% protein; and  **(2) Leucine-rich diet** containing 15% protein with 3% leucine.  **(-)** Leucine was obtained from Ajinomoto Interamericana Ind. & Com. Ltda. (Brazil) | AIN-93G | Isocaloric and isonitrogenous | Ad libitum/pair fed group |
| Ventrucci et al., 2007 | Wistar rats – Walker 256 (pregnant) | C  TB  P  CN  TBN  PN | | | immediately after the confirmation of pregnancy | 20 days | **(1) Control** AIN-93G modified diet containing 18% protein; and  **(2) Leucine-rich diet** containing 15% protein with 3% leucine.  **(-)** Leucine was obtained from Ajinomoto Interamericana Ind. & Com. Ltda. (Brazil) | AIN-93G | Isocaloric and isonitrogenous | Ad libitum/pair fed group |
| Cruz et al., 2017 | Wistar rats – Walker 256 | C  CN  TB  TBN | | | With tumor injection | 7, 14, or 21 days | **(1) Control** AIN-93G modified diet containing 18% protein; and  **(2) Leucine-rich diet** containing 15% protein with 3% leucine.  **(-)** Leucine was obtained from Ajinomoto Interamericana Ind. & Com. Ltda. (Brazil) | AIN-93G | Isocaloric and isonitrogenous | Ad libitum |
| Viana et al., 2016 | Wistar rats – Walker 256 | C  CN  TB  TBN | | | With tumor injection | 21 days | **(1) Control** AIN-93G modified diet containing 18% protein; and  **(2) Leucine-rich diet** containing 15% protein with 3% leucine.  **(-)** Leucine was obtained from Ajinomoto Interamericana Ind. & Com. Ltda. (Brazil) | AIN-93G | Isocaloric and isonitrogenous | Ad libitum |
| Gomes-Marcondes et al., 2003 | Wistar rats – Walker 256 (weanling) | C  CN  TB  TBN | | | With tumor injection | 12 days | **(1) Control** AIN-93G modified diet containing 18% protein; and  **(2) Leucine-rich diet** containing 15% protein with 3% leucine.  **(-)** Leucine was obtained from Ajinomoto Interamericana Ind. & Com. Ltda. (Brazil) | AIN-93G | Isocaloric and isonitrogenous | Ad libitum |
|  | | | | | | | | | | |
| ***β-Hydroxy-β-Methylbutyrate (HMB)*** | | | | | | | | | | |
| Smith et al., 2005 | NMRI mice – MAC16 colon | TB  TBN | | | 9 days after tumor inoculation. Just before the onset of BW loss | Animals were euthanized when the body weight loss reached 25% of their starting weight. | **(1) Control group:** PBS, daily by **oral gavage**;  **(2) HMB group:** 0.25 g per kg bodyweight, daily via **oral gavage.**  **(-) HMB was obtained from Organic Technologies Inc. (Coshocton, OH)** | Rat and mouse breeding diet (Special Diet Services) | Detail on energy and nitrogen correction not specified. | Ad libitum |
| Mirza et al., 2013 | NMRI mice – MAC16 colon | TB  TBN | | | 12 to 15 days after transplantation when the tumors became palpable and weight loss had started to occur | Animals were euthanized when the tumor ulcerated, weight loss reached 20%, or the animals became moribund. | **(1) Control group:** PBS, daily by **oral gavage**;  **(2) HMB group:** 0.25 g per kg bodyweight, daily via **oral gavage.**  **(-)** HMB was provided by Abbot Nutrition. | Rat and mouse breeding diet (Special Diet Services) | Detail on energy and nitrogen correction not specified. | Ad libitum |
| Nunes et al., 2008 | Wistar rats – Walker 256 | C  CN  TB  TBN | | | 6 weeks before tumor injection | Till 14 days after tumor inoculation (total of 8 weeks). | **(1) Control group:** 10% sucrose solution, daily via **oral gavage**;  **(2) HMB group**: 76 mg HMB per kg bodyweight in 10% sucrose solution, daily via **oral gavage**  **(-)** HMB was obtained from Metabolic Technologies Inc (Ames, IA). | Standard commercial chow (Nutrilab-CR1; Nuvital Nutrients Ltda, Curitiba-PR, Brazil) | Detail on energy and nitrogen correction not specified. | Ad libitum |
| Aversa et al., 2011 | Wistar rats – Yoshida AH-130 | C  CN  TB  TBN | | | 16 days before tumor injection | 24 days | **(1) Control** standard pelleted chow and  **(2) 4% HMB** enriched pelleted chow. | Standard pelleted chow (Mucedola, Settimo Milanese, Milan, Italy). | Detail on energy and nitrogen correction not specified. | Ad libitum |
|  | | | | | | | | | | |
| ***Glutamine*** | | | | | | | | | | |
| Fracaro et al., 2016 | Wistar rats – Walker 256 | C  CN  TB  TBN | | | With tumor injection | 14 days | **(1) Control** Standard balanced Nuvital diet (Nuvilab, Colombo, PR, Brazil);  **(2) L-glutamine-** received the standard diet with L-glutamine incorporated at a proportion of 2 g/100 g of diet.  **(-)** L-glutamine was obtained from Deg (Sao Paulo, SP, Brazil) | Standard balanced Nuvital diet (Nuvilab, Colombo, PR, Brazil) | Detail on energy and nitrogen correction not specified. | Ad libitum |
| Martins et al., 2016 | Wistar rats – Walker 256 | C  CN  TB  TBN | | | With tumor injection | 10 days | **(1) Control** Standard balanced Nuvital diet (Nuvilab, Colombo, PR, Brazil);  **(2) L-glutamine-** received the standard diet with L-glutamine incorporated at a proportion of 2 g/100 g of diet.  **(-)** L-glutamine was obtained from Fagron of Brazil Pharma Ltda (Sao Paulo, SP, Brazil) | Standard balanced Nuvital diet (Nuvilab, Colombo, PR, Brazil) | Detail on energy and nitrogen correction not specified. | Ad libitum |
| Martins et al., 2017 | Wistar rats – Walker 256 | C  CN  TB  TBN | | | With tumor injection | 10 days | **1) Control** Standard balanced Nuvital diet (Nuvilab, Colombo, PR, Brazil);  **(2) L-glutamine-** received the standard diet with L-glutamine incorporated at a proportion of 2 g/100 g of diet.  **(-)** L-glutamine was obtained from Fagron of Brazil Pharma Ltda (Sao Paulo, SP, Brazil) | Standard balanced Nuvital diet (Nuvilab, Colombo, PR, Brazil) | Detail on energy and nitrogen correction not specified. | Ad libitum |
| Vincentini et al., 2016 | Wistar rats – Walker 256 | C  CN  TB  TBN | | | With tumor injection | 14 days | **1) Control** Standard balanced Nuvital diet (Nuvilab, Colombo, PR, Brazil);  **(2) L-glutamine-** received the standard diet with L-glutamine incorporated at a proportion of 2 g/100 g of diet.  **(-)** L-glutamine was obtained from Deg (Sao Paulo, SP, Brazil) | Standard balanced Nuvital diet (Nuvilab, Colombo, PR, Brazil) | Detail on energy and nitrogen correction not specified. | Ad libitum |
| Vincentini et al., 2017 | Wistar rats – Walker 256 | C  CN  TB  TBN | | | With tumor injection | 14 days | **1) Control** Standard balanced Nuvital diet (Nuvilab, Colombo, PR, Brazil);  **(2) L-glutamine-** received the standard diet with L-glutamine incorporated at a proportion of 2 g/100 g of diet.  **(-)** L-glutamine was obtained from Deg (Sao Paulo, SP, Brazil) | Standard balanced Nuvital diet (Nuvilab, Colombo, PR, Brazil) | Detail on energy and nitrogen correction not specified. | Ad libitum |
|  | | | | | | | | | | |
| ***Glycine*** | | | | | | | | | | |
| Ham et al., 2014 | CD2F1 mice – Colon 26 | | C  TB  TBN | With tumor injection | | 21 days | **(1) Control:** subcutaneous injection of saline  **(2) Glycine**: 1 g per kg bodyweight of **glycine** in PBS once daily via **subcutaneous injections.** | Standard laboratory chow (not further specified). | Detail on energy and nitrogen correction not specified. | Ad libitum |
|  | | | | | | | | | | |
| ***Ketogenic High fat diets*** | | | | | | | | | | |
| Tisdale et al., 1987 | NMRI mice – MAC16 colon | C  CN  TB  TBN | | | 8 days after tumor transplantation | 20 days | **(1) Control diet**: standard laboratory chow; 11.5% total kcal as fat;  **(2) Medium-chain triglyceride (MCT) diet**: modified standard laboratory chow with 80% total kcal from MCT. | Standard laboratory chow (Pilsbury, Birmingham, United Kingdom) | Isocaloric and isonitrogenous | Ad libitum |
| Beck et al., 1989 | NMRI mice – MAC16 colon | C  TB  TBN | | | 14 days after tumor transplantation, at which time the tumors were palpable but weight loss had not occurred. | 9 days | **(1) Control diet**: standard laboratory chow; 11.5% total kcal as fat;  **(2) MCT diet**: modified standard laboratory chow with 80% total kcal from MCT. | Standard laboratory chow (Pilsbury, Birmingham, United Kingdom) | Isocaloric and isonitrogenous | Ad libitum |
| Nakamura et al., 2018 | CD2F1 mice – Colon 26 | C  TB  TBN | | | With tumor injection | Until moribund condition max. 21 days. | **(1) Control diet** is AIN-93G (64% C, 20% P, and 16% L);  **(2) ketogenic formula**, Ketonformula 817-B (Meij Co.; KF 5% C, 8% P, and 87% L). | AIN-93G | Isocaloric, not isonitrogenous | Ad libitum |
|  | | | | | | | | | | |
| ***Polyunsaturated fatty acids (PUFAs)*** | | | | | | | | | | |
| Tisdale et al., 1990 | NMRI mice – MAC16 colon | C  TB  TBN | | | 12-14 days after tumor transplantation, at which time the tumors were palpable but weight loss had not occurred. | Animals were euthenized when the tumor ulcerated, weight loss reached 6 to 7 g, or the animals became moribund | **(1) Control diet**: standard laboratory chow; 11.5% total kcal as fat;  **(2) Fish oil (FO) diet**: modified standard laboratory chow with 5%, 10%, 25% or 50% fish oil.  **(-)** Fish oil was a product of the Q.P. corporation (Shibuya, Japan) | Standard laboratory chow (Pilsbury, Birmingham, United Kingdom) | Isocaloric and isonitrogenous | Ad libitum |
| Pizato et al., 2005 | Wistar rats – Walker 256 | C  TB  TBN | | | 8 weeks before tumor injection. | Till 14 days after tumor inoculation (total of 10 weeks). | **(1) Control group:** standard chow diet, 62% total kcal carbohydrates, 29% total kcal protein, 9% total kcal fat;  **(2) High‐fat FO diet:** modified standard laboratory chow, 49% total kcal fat, 29% total kcal carbohydrates, 22% total kcal protein; 66% of fat as substituted as FO. | Standard chow diet (Nuvital CR-1, Curitiba, Brazil) | Isonitrogenous, not isocaloric | Ad libitum |
| Coelho et al., 2012 | Wistar rats – Walker 256 | C  CN  TB  TBN | | | 10 weeks before tumor injection. | Till 14 days after tumor inoculation (total of 12 weeks). | **1) Control group:** 1 g of coconut oil per kg bodyweight, daily via **oral gavage.**  **(2) FO group:** 1 g of fish oil per kg bodyweight, daily via **oral gavage.**  This dose represents approximately 1.25 % (w/w) of the diet.  **(-)** The FO used was a mixed marine triacylglycerol preparation containing 180 g eicosapentaenoic acid (EPA) and 120 g docosahexaenoic acid (DHA) per kg. | Standard chow diet (Nuvital CR-1, Curitiba, Brazil) | Detail on energy and nitrogen correction not specified. | Ad libitum |
| Togni et al., 2003 | Wistar rats – Walker 256 | C_1_  C_2_  CN  TB_1_  TB_2_  TBN | | | Lifelong supplementation: Female Wistar rats were supplemented with FO prior to mating and then throughout pregnancy and gestation, and then the male offspring were supplemented from weaning until 90 days of age. Then they were inoculated subcutaneously with tumor cells. | Till 14 days after tumor inoculation. | **(1) Control group:** standard chow diet  **(2) CO group:** 1 g of coconut oil per kg bodyweight, daily via **oral gavage.**  **(3) FO group:** 1 g of fish oil per kg bodyweight, daily via **oral gavage.**  **(-)** The FO used was a mixed marine triacylglycerol preparation containing 180 g eicosapentaenoic acid (EPA) and 120 g docosahexaenoic acid (DHA) per kg.  **(-)** Fish oil was obtained from MaxEpa (Seven Seas, Hull, UK) | Standard chow diet | Detail on energy and nitrogen correction not specified. | Ad libitum |
| Fernandez et al., 2004 | Wistar rats – Walker 256 | C  CN  TB  TBN | | | Lifelong supplementation (see Togni et al., 2003) | Till 14 days after tumor inoculation | **FO group:** 1 g of fish oil per kg bodyweight, daily via **oral gavage.** This dose represents approximately 1.25 % (w/w) of the diet.  **(-)** The FO used was a mixed marine triacylglycerol preparation containing 180 g eicosapentaenoic acid (EPA) and 120 g docosahexaenoic acid (DHA) per kg. | Standard chow diet (Nuvital CR-1, Curitiba, Brazil) | Detail on energy and nitrogen correction not specified. | Ad libitum |
| Dumas et al., 2010 | BDIX rats - DHD/K12 cells | C  TB  P  CN  TBN  PN | | | 6 weeks before tumor injection. | Till food intake was reduced by 25% for 11 consecutive days. | **(1) Control group:** standard laboratory diet  **(2) FO diet:** modified standard diet, incorporated with 5% FO.  **(-)** Fish oil was obtained from Phosphotech (St. Herblain, France) | Standard laboratory diet (National Institute of Research on Agronomics, INRA, Jouyen-Josas, France). | Isocaloric and isonitrogenous | Ad libitum/pair-fed group |
| Iagher et al., 2011 | Wistar rats – Walker 256 | C_1_  C_2_  CN  TB_1_  TB_2_  TBN | | | 7 weeks before tumor injection. | Till 14 days after tumor inoculation (total of 9 weeks). | **(1) Control group:** standard chow diet  **(2) CO group:** 1 g of coconut oil per kg bodyweight, daily via **oral gavage.**  **(3) SLO group:** 1 g of shark liver oil per kg bodyweight, daily via **oral gavage.**  **(-) SLO** was obtained from Naturalis Alimentos Naturais Ltda (Sao Paulo, Brazil) | Standard chow diet (Nuvital CR-1, Curitiba, Brazil) | Detail on energy and nitrogen correction not specified. | Ad libitum |
| Iagher et al., 2013 | Wistar rats – Walker 256 | C  CN_1_  CN_2_  TB  TBN_1_  TBN_2_ | | | 8 weeks before tumor injection. | Till 14 days after tumor inoculation (total of 10 weeks). | **(1) Control group:** standard chow diet  **(2) FO group:** 1 g of fish oil per kg bodyweight, daily via **oral gavage.**  **(3) SLO group:** 1 g of shark liver oil per kg bodyweight, daily via **oral gavage.** | Standard chow diet (Nuvital CR-1, Curitiba, Brazil) | Detail on energy and nitrogen correction not specified. | Ad libitum |
| Beck et al., 1991 | NMRI mice – MAC16 colon | C  CN  TB  TBN | | | 10 to 12 days after transplantation when the tumors became palpable and weight loss had started to occur. | Max 9 days. Animals were euthanized when the tumor ulcerated, weight loss reached 25 to 30%, the tumor weight reached 10% of the host body weight, or the animals became moribund. | **(1) Control group:** solvent (liquid paraffin:water, 2:1), daily by **oral gavage**;  **(2) EPA** **group:** 1.25 to 2.5 g of EPA per kg bodyweight, daily by **oral gavage.** | Standard laboratory chow (Pilsbury, Birmingham, United Kingdom) | Detail on energy and nitrogen correction not specified. | Ad libitum |
| Tisdale et al., 1991 | NMRI mice – MAC16 colon | TB  TBN_1_  TBN_2_  TBN_3_ | | | 10 to 12 days after transplantation when the tumors became palpable and weight loss had started to occur. | 5 days | **(1) Control group:** 100 uL 0.9% saline, daily by oral gavage;  **(2) EPA group:** 100 mg EPA, daily by oral gavage;  **(3) DHA group:** 100 mg DHA, daily by oral gavage;  **(4) Linoleic acid group:** 100 mg linoleic acid, daily by oral gavage.  **(-)** Linoleic acid and DHA (both 99% pure) were obtained from the Sigma Chemical Co. (Poole, U.K.), and EPA (95% pure) was obtained from Peninsula Laboratories Europe Ltd (Merseyside, U.K.). | Standard laboratory chow (Pilsbury, Birmingham, United Kingdom) | Detail on energy and nitrogen correction not specified. | Ad libitum |
| Whitehouse et al., 2001 | NMRI mice – MAC16 colon | C  CN  TB  TBN | | | 10 to 12 days after transplantation when the tumors became palpable and weight loss had started to occur. | 0, 24 and 48 hours | **(1) Control group:** olive oil, daily by **oral gavage**;  **(2) EPA** **group:** 0.5 and 2.5 g of EPA per kg bodyweight, daily by **oral gavage.** | Standard laboratory chow (Special Diet Services, Witham, UK). | Detail on energy and nitrogen correction not specified. | Ad libitum |
| Smith et al., 2004 | NMRI mice – MAC16 colon | C  TB  TBN | | | After 5% BW loss was reached. (Weight loss was evident 10–12 days after transplantation) | 4 days | **(1) Control group:** olive oil, twice daily by **oral gavage**;  **(2) EPA** **group:** 1 g of EPA per kg bodyweight, twice daily by **oral gavage.** | Economy rodent breeder diet (Special Diet Services, Essex, UK). | Detail on energy and nitrogen correction not specified. | Ad libitum |
| Du et al., 2015 | Kunming mice – S180 cells | C  TB  TBN | | | 24 hours after tumor injection | 14 days | **(1) Control group:** 0.85% normal saline, daily by **oral gavage**;  **(2) EPA-PL** **group:** 100 mg of EPA per kg bodyweight, daily by **oral gavage.** | Standard chow diet (Harlan Teklad LM-485). | Detail on energy and nitrogen correction not specified. | Ad libitum |
| Fini et al., 2010 | Apc(Min/+) | C  CN_1_  CN_2_  TB  TBN_1_  TBN_2_ | | | Start 6 weeks old | 12 weeks | **(1) Control diet:** modified AIN‐93G diet, corn oil substituted for soybean oil;  **(2) EPA diet:** modified AIN‐93G diet, soybean oil substituted for EPA free fatty acids at 2.5% weight/weight (w/w);  **(3) EPA diet:** modified AIN‐93G diet, soybean oil substituted for EPA free fatty acids at 5% w/w.  **(-)** EPA was obtained from ALFA, SLA Pharma AG. | AIN-93G (Research Diets) | Isocaloric and isonitrogenous | Controlled |
| Jho et al., 2002 | Fisher 344 rats - MCA tumor | TB_1_  TB_2_  TBN | | | 13 days after tumor injection. | 16 days | **(1) Control: oral gavage** isovolemic 5.0 g/kg of corn oil (isocaloric) combined with 10 IU vitamin E/g fat,  **(2) Control:** isovolumic but non-isocaloric normal saline combined with 10 IU vitamin E/g;  **(2) EPA:** **oral gavage** twice daily, providing 5.0 g/kg per day of EPA with 10 IU vitamin E/g fat. | Undefined | Isocaloric and isonitrogenous | Controlled |
| Dagnelie et al., 1994 | Fisher F1 hybrid rats - MATLy-Lu prostate tumor cells | C  CN  TB  TBN | | | 7 days after tumor injection. | 14 days | **(1) Control diet:** standard laboratory chow containing 50% total kcal carbohydrates, 20% total kcal protein, 11,5 % total kcal fat.  (**2) FO diet**: semi purified diet, containing 50% total kcal coming from fish oil, which substituted for carbohydrates. | Standard laboratory chow (Pilsbury, Birmingham, United Kingdom) | Isocaloric and isonitrogenous | Ad libitum |
|  | | | | | | | | | | |
| ***Conjugated linoleic acid (CLA)*** | | | | | | | | | | |
| Graves et al., 2005 | CD2F1 mice - Colon 26 | C  CN  TB  TBN | | | With tumor injection and 2 weeks before tumor injection. | 5 weeks (Till 21 days after tumor injection) | **(1) Control diet:** pulverized rodent chow;  (**2) CLA diet:** pulverized rodent chow supplemented with 0.5% CLA. | Standard laboratory chow | Detail on energy and nitrogen correction not specified. | Ad libitum |
| McCarthy et al., 2006 | C57Bl/6 mice – LLC and B16 tumor cells | C  CN  TB  TBN | | | With tumor injection. | 17 days | **(1) Control diet:** pulverized rodent chow;  **(2) CLA diet:** pulverized rodent chow supplemented with 0.5% CLA.  **(-)** CLA was obtained from Loders CrokLaan (Channahon, IL) | Standard laboratory chow | Detail on energy and nitrogen correction not specified. | Ad libitum |
| Tian et al., 2011 | CD2F1 mice - Colon 26 | C  TB  TBN  P | | | With tumor injection. | 20% difference in body weight between PBS and Tumor groups (day 17 post‐inoculation) | **(1) Control diet:** 7% soybean oil (PBS, Tumor and Pair‐fed groups);  **(2) CLA diet:** 6% soybean oil and 1% c9t11‐CLA free fatty acids (C9t11‐CLA FFA)  **(-)** CLA was obtained from Lipid Nutrition (Wormerveer, the Netherlands) | AIN-93G (Research Diets, Brunswick, NJ, USA) | Isocaloric and isonitrogenous | Controlled (pair-fed) |
| Goncalves et al., 2019 | Wistar rats – Walker 256 | C_1_  C_2_  CN  TB_1_  TB_2_  TBN | | | With tumor injection. | 14 days | **(1) Control group:** 0.5 ml of 0.9% saline, daily by **oral gavage**;  **(2) Control group:** 0.5 ml of sunflower oil, daily by **oral gavage**;  **(3) CLA** **group:** 0.5 ml (2% w:w of total food intake) of CLA (Tonalin®), daily by **oral gavage.** | Standard balanced Nuvital diet (Nuvilab, Colombo, PR, Brazil) | Detail on energy and nitrogen correction not specified. | Ad libitum |
|  | | | | | | | | | | |
| ***Carnitine*** | | | | | | | | | | |
| Busquets et al., 2012 | Wistar rats – Yoshida AH-130 | TB  TBN | | | With tumor injection. | 7 days | **(1) Control:** corresponding volume of cornoil, daily by **oral gavage**;  **(2) L-Carnitine:** 1 g of L-carnitine per kg bodyweight, daily by **oral gavage.**  **(-)** L-Carnitine was obtained from Sigma (Tau, Spain) | Standard laboratoty chow (Harlan Teklad Global Diet 2014) | N.A. | Ad libitum |
| Busquets et al., 2020 | Wistar rats – Yoshida AH-130 | TB  TBN | | | With tumor injection. | 7 days | **(1) Control:** corresponding volume of cornoil, daily by **oral gavage**;  **(2) L-Carnitine:** 1 g of L-carnitine per kg bodyweight, daily by **oral gavage.**  **(-)** L-Carnitine was obtained from Sigma (Tau, Spain) | Undefined | N.A. | Ad libitum |
| Liu et al., 2011 | BALB/c mice - Colon 26 | C  TB  TBN | | | 12 days after tumor injection. | 7 days | **(1) Control:** 2 ml of 0.9% saline, daily by **oral gavage**;  **(2) L-Carnitine:** 4.5 mg of L-carnitine per kg of bodyweight, daily by **oral gavage**;  **(3) L-Carnitine:** 18 mg of L-carnitine per kg of bodyweight, daily by **oral gavage**;  **(-)** L-Carnitine was obtained from Sigma-Aldrich (St. Louis, MO, USA) | Undefined | N.A. | Ad libitum |
| Silverio et al., 2012 | Wistar rats – Walker 256 | C  CN  TB  TBN | | | 14 days before tumor injection. | 28 days (Until 14 after tumor injection). | **(1) Control:** 1 ml of 0.9% saline, daily by **oral gavage**;  **(2) L-Carnitine:** 1 g of L-carnitine per kg of bodyweight, daily by **oral gavage**.  **(-)** L-Carnitine was obtained from ICN-USA | Standard laboratory chow (NUVILAB 1, Nuvital, Brazil) | N.A. | Ad libitum |
|  | | | | | | | | | | |
| ***Creatine*** | | | | | | | | | | |
| Campos-Ferraz et al., 2016 | Wistar rats – Walker 256 | C  TB  TBN | | | 7 days after tumor injection. | 15 and 33 days | **(1) Control:** 4 ml of PBS, daily by **oral gavage**;  **(2) Creatine:** 4 ml of PBS containing 300 mg of creatine per kg of bodyweight, daily by **oral gavage**. | Standard laboratory chow | N.A. | Ad libitum |
| Cella et al., 2020 | Wistar rats – Walker 256 | C  TB  TBN | | | With tumor injection. | 21 days | **Creatine:** 8 g/l creatine monohydrate in their drinking water (1.0±0.1 g/kg/day) | Standard chow diet (Nuvital CR-1, Curitiba, Brazil) | N.A. | Ad libitum |
| Deminice et al., 2015 | Wistar rats – Walker 256 | C  TB  TBN | | | 11 days before tumor injection. | 21 days (10 days after tumor injection) | **Creatine:** 8 g/l creatine monohydrate in their drinking water. | Standard chow diet (Nuvital CR-1, Curitiba, Brazil) | N.A. | Ad libitum |
|  | | | | | | | | | | |
| ***Flavonoids*** | | | | | | | | | | |
| Velazquez et al., 2014 | C57Bl/6J and Apc(Min/+) | C  CN  TB  TBN | | | Start at 15 weeks old. | 21 days | **(1) Quercetin:** 25 mg/kg (Nutravail) mixed with orange-flavored Tang (Kraft Foods), daily via oral gavage;  **(2) Control:** an equal volume of vehicle solution mix of Tang juice and water alone, daily via oral gavage. | Standard rodent unpurified diet (HarlanTeklad Rodent Diet, no. 8604) | Detail on energy and nitrogen correction not specified. | Ad libitum |
| Camargo et al., 2011 | Wistar rats – Walker 256 | TB  TBN | | | 3h before tumor injection. | 15 days | **Quercetin** was dissolved in 0.3 mL mineral oil (Nujol) and **intraperitoneal** administered daily.  **(-)** Quercetin was obtained from Sigma Chemical Co. (St. Louis, MO, USA) | Undefined | Detail on energy and nitrogen correction not specified. | Ad libitum |
| Wang et al., 2011 | C57Bl/6 mice – LLC cells | TB  TBN_1_  TBN_2_ | | | Preventative: 12 days before tumor injection.  Curative: with tumor injection | 32-34 days | **(1) Control:** water, daily by **oral gavage**;  **(2) EGCG low:** 0.2 mg of EGCG, daily by **oral gavage**;  **(3) EGCG high:** 0.6 mg EGCG, daily by **oral gavage**;  **(-)** EGCG was obtained from Sigma Chemical Co. (St. Louis, MO, USA) | Standard laboratory chow (laboratory rodent diet, labdiet 5001, USA) | Detail on energy and nitrogen correction not specified. | Ad libitum |
| Hirasaka et al., 2016 | C57Bl/6 mice – LLC cells | C  CN  TB  TBN | | | With tumor injection. | 21 days | **(1) Control diet:** standard chow, AIN-93M;  **(2) Isoflavone diet:** the alpha-starch content of the isoflavone diet was reduced to adjust for the composition of other nutrients, and comprised a normal diet (based on AIN-93M) mixed with soya flavone HG (0,4% w/w; Fuji Oil Co.) | AIN-93M (Oriental Yeast Co., Ltd., Tokyo, Japan). | Isocaloric and isonitrogenous | Ad libitum |
| Yoshimura et al., 2018 | C57Bl/6 mice – LLC cells | C  CN  TB  TBN | | | With tumor injection. | 21 days | **(1) control diet**: AIN-93M;  **(2) morin diet:** The a-starch content of the morin diet was reduced to adjust for the composition of other nutrients, and comprised a normal diet (based on AIN-93M) mixed with morin (0.1% w/w)  **(-)** Morin was obtained from Sigma Aldrich (St. Louis, MO, USA) | AIN-93M (Oriental Yeast Co., Ltd., Tokyo, Japan). | Isocaloric and isonitrogenous | Ad libitum |
|  | | | | | | | | | | |
| ***Resveratrol*** | | | | | | | | | | |
| Wyke et al., 2004 | NMRI mice – MAC16 colon | TB  TBN | | | After 5% BW loss was reached. (Weight loss was evident 10–12 days after transplantation). | 5 days | **(1) Resveratrol‐treated:** Daily i.p. dose of resveratrol (1 mg/kgBW);  **(2) Control:** solvent (DMSO : PBS (1 : 20)) i.p. dose.  **(-)** Resveratrol was obtained from Biomol Research Laboratories Inc. (PA, USA) | Rat and mouse breeding diet (Special Diet Services, Witham, UK) | N.A. | Ad libitum |
| Shadfar et al., 2011 | CD2F1 mice - Colon 26 | C  CN  TB  TBN | | | 6 days after tumor injection. | 11 days | **(1) Control** 250 µl of vehicle, daily by oral gavage;  **(2) Resveratrol** 100 mg/kg, 200 mg/kg, or 500 mg/kg of resveratrol, daily by oral gavage.  **(-)** Resveratrol was obtained from Cayman Chemical (Ann Arbor, MI, USA) | Undefined | N.A. | Ad libitum |
| Busquets et al., 2007 | C57Bl/6 - LLC and  Wistar rats - Yoshida AH-130 | C  CN  TB  TBN | | | With tumor injection. | C57Bl/6 mice: 15 days;  Wistar rats: 7 days. | C57Bl/6 mice diets:  **(1) Resveratrol‐treated:** i.p. dose of resveratrol (5 or 25 mg/kg BW);  **(2) Control:** i.p. dose of saline (5 or 25 mg/kg BW).  Wistar rats diets:  **(1) Resveratrol‐treated:** Daily i.p. dose of resveratrol (1 mg/kgBW);  **(2) Control:** saline i.p. dose (1 mg/kgBW). | Undefined | N.A. | Ad libitum |
|  | | | | | | | | | | |
| ***Prebiotic non-digestible oligosaccharides*** | | | | | | | | | | |
| Gorselink et al., 2006 | CD2F1 mice - Colon 26 | C  TB  TBN | | | With tumor injection. | 20 days | Experiment 1:  **(1) Control:** AIN-93M;  **(2)** **GOS/FOS diet:** The modified AIN-93 M diet consisted of 51% galacto-oligosaccharides (GOS) and fructo-polysaccharides (9:1), 19% maltodextrin, 16% lactose and 14% glucose in experiment 1.  Experiment 2:  **(1) Control:** AIN-93M;  **(2)** **GOS/GOS diet:** The FOS was replaced by additional GOS. | AIN-93M diet (Research Diets) | Detail on energy and nitrogen correction not specified. | Ad libitum |
| Bindels et al., 2015 | BALB/c - BaF3(Bcr-Abl) | C  TB  TBN_1_  TBN_2_ | | | 1 day after tumor injection. | 15 days | **(1) Control:** AIN93M;  **(2) Inulin:** AIN93M supplemented with inulin (5% INU w/w); and  **(3) POS:** AIN93M diet supplemented with POS (5% POS w/w). | AIN-93M (Research diets, New Brunswick, NJ, USA) | Detail on energy and nitrogen correction not specified. | Ad libitum |

C: Control group, CN: control group + nutrition intervention, TB: tumor-bearing group, TBN: tumor-bearing group + nutrition intervention, P: pair-fed group, PN: pair-fed + nutrition intervention, and N.A.: Not Applicable.

A literature search was performed in PubMed. Papers published in the last 35 years were included (1985-2020). The search string was composed of three search components (SCs): SC1 nutritional intervention, SC2 cancer [and] cachexia, and SC3 laboratory animals. For each of the critical SCs, a separate search string was developed. Each component-specific search string included an extensive collection of appropriate search terms, including Medical Subject Heading and free-text terms. For SC3, a PubMed search filter for primary animal studies was used (1). In a final search, all separately developed search strings for each SC were combined to retrieve the potentially relevant studies. Papers were excluded if (i) studies did not include a dietary intervention, (ii) studies did include a pharmacological intervention, (iii) studies did not include tumor-induced cachexia models, (iv) studies did not describe the conventional phenotype of cachexia, (v) human intervention studies, and (vi) review papers.

1. Hooijmans CR, Tillema A, Leenaars M, Ritskes-Hoitinga M. Enhancing search efficiency by means of a search filter for finding all studies on animal experimentation in PubMed. Laboratory Animals. 2010;44(3):170-5 DOI: 10.1258/la.2010.009117.
